# Comments: "Effect of long-term negative energy on appetite hormone levels in individuals with prediabetes and diabetes"

**DOI:** 10.1590/1806-9282.20250818

**Published:** 2025-12-08

**Authors:** Sami A. Gabr

**Affiliations:** 1 Mansoura University, Faculty of Medicine, Department of Medical Microbiology and Immunology, Genetics of Microbiology Unit – Mansoura, Egypt.

## COMMENTARY

Appetite-regulating hormones play a pivotal role in energy homeostasis, influencing food intake and body weight. In individuals with prediabetes and T2D, disruptions in the balance of these hormones contribute to the pathophysiology of these conditions^1-4^. Interventions such as calorie restriction and exercise aim to restore this balance, thereby improving metabolic health. However, the effects of long-term negative energy balance on appetite hormones in these populations remain under investigation^5-8^. Feeding behavior is integrated within a wide variety of eating behaviors, which depend on psychosocial, biological, and environmental factors. Appetite hormones such as ghrelin, leptin, GLP-1, and PYY, are some of the most important body hormones that regulate appetite and prevent more appetite-related diseases such as obesity and diabetes^9-13^. Ghrelin and leptin are key hormones that regulate appetite, food intake, and energy metabolism^14,15^. Ghrelin, known as the "hunger hormone," stimulates appetite and promotes fat storage. Studies have shown that in individuals with T2D, long-term calorie restriction and exercise can lead to increased ghrelin levels, potentially enhancing appetite regulation. Also, leptin, produced by adipose tissue, signals satiety and regulates energy balance^13,15-18^. In T2D, leptin resistance is common, leading to impaired appetite suppression^13,15-18^. Thus, long-term interventions of negative energy balance may improve leptin sensitivity, aiding in better appetite control. Moreover, GLP-1 and PYY, gut-derived hormones, promote satiety and insulin secretion. Evidence suggests that long-term negative energy balance can enhance GLP-1 and PYY levels, improving appetite regulation and glycemic control in T2D^19,20^.

Research in genetics suggests that genetic variants of both hormones are associated with complex forms of eating behavior, such as a preference for palatable food, making individuals susceptible to the modern obesogenic environment. Thus, the understanding of the mechanisms relating to the action of these appetite hormones is relevant since it could impact the objectives of pharmacological or behavioral interventions for their treatment^16,21^.

Thus, from the current research work, it was highlighted that differential responses of appetite hormones to long-term negative energy balance in prediabetes and T2D underscore the need for tailored interventions. While T2D individuals may experience beneficial hormonal changes, prediabetic individuals might not exhibit significant alterations, suggesting the necessity for early intervention to prevent progression to T2D. Furthermore, factors such as obesity, insulin resistance, and medication use can influence hormonal responses, highlighting the complexity of managing appetite regulation in these populations^22^.

Moreover, disorder in appetite hormones as a result of long-term negative energy balance and related weight loss can worsen the conditions of patients with cardiovascular and cancer diseases. Research indicates that individuals with heart disease may experience altered leptin and ghrelin levels, leading to an increased appetite for energy-dense foods^23-27^. Furthermore, dysregulated energy balance can exacerbate inflammation and insulin resistance, which are risk factors for cardiovascular conditions^28-31^. In addition, cancer patients undergoing long-term negative energy balance, especially during chemotherapy or other treatments, often experience changes in appetite hormones, leading to issues such as cachexia (muscle wasting) or severe weight loss^32^. Reduced levels of leptin and elevated ghrelin can promote further weight loss and anorexia, which complicates nutritional management and disease progression^33^. This dysregulation of appetite hormones can worsen the prognosis by diminishing the body's ability to maintain adequate nutrition and support immune function during cancer treatment^28-31,34-36^. Thus, maintaining a balanced energy intake may be crucial in managing both heart and cancer diseases via appetite hormone regulation.

Finally, long-term negative energy balance disrupts the regulation of appetite hormones, which can have far-reaching implications in metabolic conditions such as prediabetes and diabetes. This effect is not limited to metabolic diseases but extends to other chronic conditions, such as heart disease and cancer, where altered appetite regulation can worsen outcomes. For individuals with these diseases, careful attention to energy balance and hormone regulation may be essential for managing symptoms and improving overall health.

So, future research perspectives with long-term periods should focus on assessing the long-term effects of sustained calorie restriction and exercise on appetite hormones across different stages of metabolic dysfunction, exploring the molecular pathways linking negative energy balance to hormonal changes, including the role of the hypothalamus and gut–brain axis, and developing individualized treatment plans based on hormonal profiles and genetic predispositions to optimize outcomes.

Thus, the following recommendations were required: it was recommended that an early intervention to implement calorie restriction and exercise programs at the prediabetic stage to prevent progression to T2D and progression of other subsidiary risk factors associated with chronic heart and cancer diseases; then, a comprehensive monitoring to regularly assess appetite hormone levels to tailor interventions effectively; and finally, education for individuals on the importance of lifestyle modifications was appreciated in managing appetite and preventing metabolic diseases. This might proceed by incorporating dietary modifications, physical activity, and behavioral therapies to address the multifaceted nature of appetite regulation in patients as well as healthy humans.

## Data Availability

The datasets generated and/or analyzed during the current study are available from the corresponding author upon reasonable request.
